# Lipemia in the Plasma Sample Affects Fentanyl Measurements by Means of HPLC-MS^2^ after Liquid-Liquid Extraction

**DOI:** 10.3390/molecules26154514

**Published:** 2021-07-27

**Authors:** Marta Tikhomirov, Tomasz Śniegocki, Błażej Poźniak

**Affiliations:** 1 Department of Pharmacology and Toxicology, Faculty of Veterinary Medicine, Wroclaw University of Environmental and Life Sciences, 50-375 Wrocław, Poland; zaklad.toksykologii@upwr.edu.pl; 2 Department of Pharmacology and Toxicology, National Veterinary Research Institute, 24-100 Puławy, Poland; sniego@piwet.pulawy.pl

**Keywords:** fentanyl, lipemia, mass spectrometry, high performance liquid chromatography tandem mass spectrometry

## Abstract

Examination of fentanyl levels is frequently performed in certain scientific evaluations and forensic toxicology. It often involves the collection of very variable blood samples, including lipemic plasma or serum. To date, many works have reported the methods for fentanyl detection, but none of them have provided information about the impact on the assay performance caused by an excessive amount of lipids. This aspect may be, however, very important for highly lipophilic drugs like fentanyl. To address this issue, we developed the liquid chromatography method with mass spectrometry detection and utilized it to investigate the impact of lipids presence in rabbit plasma on the analytical method performance and validation. The validation procedure, conducted for normal plasma and lipemic plasma separately, resulted in good selectivity, sensitivity and linearity. The limits of detection and quantification were comparable between the two matrices, being slightly lower in normal plasma (0.005 and 0.015 µg/L) than in lipemic plasma (0.008 and 0.020 µg/L). Liquid–liquid extraction provided a low matrix effect regardless of the lipid levels in the samples (<10%), but pronounced differences were found in the recovery and accuracy. In the normal plasma, this parameter was stable and high (around 100%), but in the lipemic matrix, much more variable and less efficient results were obtained. Nevertheless, this difference had no impact on repeatability and reproducibility. In the present work, we provided reliable, convenient and sensitive method for fentanyl detection in the normal and lipemic rabbit plasma. However, construction of two separate validation curves was necessary to provide adequate results since the liquid-liquid extraction was utilized. Therefore, special attention should be paid during fentanyl quantification that involves lipemic plasma samples purified by this technique.

## 1. Introduction

Sample turbidity due to accumulation of lipoprotein particles is a relatively frequently reported feature in blood samples collected for analysis [1]. This condition, often described as lipemia, may be of special concern for certain analytical procedures [1]. Lipemia can be caused by several factors and is usually observed if a too short time elapsed between the last meal consumed by a patient and blood sampling [2]. Additionally, several pathophysiological conditions, such as diabetes mellitus [3], multiple myeloma [4], acute pancreatitis [5] or kidney failure [6] can cause persisting lipemia regardless of fasting. Therefore, in some cases, collection of lipemic samples is unavoidable due to the patient’s condition and emergency of diagnostics or even treatment, as some patients may require direct lipid infusions right before or during the sampling [7,8]. Such samples often require sophisticated analysis regardless of the lipid content. The major problem faced by a laboratory is how to analyze such a sample without the risk of lipid interference with an assay performance. Depending on the analytical approach, different protocols may be optimal in different cases. In commonly performed immune or coulometric assays, the potential error caused by lipids may depend on their levels. At some concentrations, no special actions are needed but after a certain threshold is passed, the lipids can interfere with the results [9]. In such a situation, a typical approach is to remove excessive lipids by ultracentrifugation. The procedure allows for easy and rapid destabilization and sequestration of the lipid layer. Despite its convenience, there are some situations where the described protocol may, however, affect the results. An example can be a drug assay, as explained below. Irrespective of the selected detection method, pretreatment and purification of the sample can introduce high bias if conducted inappropriately. This can be especially true for difficult and atypical samples such as lipemic plasma, serum or blood. During a drug analysis, ultracentrifugation of the sample can result in sequestration of not only the lipids but also of the drug itself [10]. Due to this, ultracentrifugation of the sample is a seldom-seen solution for the drug analysis from the lipemic matrix. A much more often reported approach is preparation and evaluation of a detection procedure where lipemia is one of the tested factors, and typically, where its negative impact is excluded [11,12,13,14,15]. Despite very rarely reported interferences with high performance liquid chromatography (HPLC), i.e., one of the most common analytical techniques for drug assessment, some authors described certain issues related to the lipemic samples for some drugs. For example, lipid-rich matrix caused instability of olanzapine assay [16], and another work reported that lipemia was even responsible for false-negative diagnosis in a patient poisoned with vitamin D [17]. However, none of these reports concerned fentanyl (FEN). This highly potent, synthetic opioid drug is widely used to provide analgesia for the patients [18]. At the same time, it is known for its illicit use and grave potential consequences in case of overdose [19]. High prevalence of the drug in forensic toxicology cases called for effective methods for its detection in biological samples. A number of methods developed for FEN are reported in available literature [20,21,22,23,24,25]. Most of them use HPLC coupled with tandem mass spectrometry (HPLC-MS^2^) that provides specificity, accuracy and sensitivity during the drug analysis as well as short analytical run time, relatively small sample size required and simplicity of the sample preparation [26]. However, to the best of our knowledge, there has been no report published on the potential impact of lipemia on FEN assay performance. As the level of this opioid is often measured in therapeutic drug monitoring or in emergency unit toxicological cases [27,28,29], high variability of the matrices has to be taken into consideration. This also includes potential evaluation of lipemic plasma or serum samples. During mass spectrometry analysis, lipid content in the sample can potentially affect the analysis on several levels. The efficacy of an extraction procedure can be impacted by lipids to a different degree depending on the purification method selected. In this aspect, liquid-liquid extraction presents different challenges than solid phase extraction. The first one may provide insufficient purification of the sample, causing transmission of the lipids to the HPLC equipment. On the other hand, in some circumstances, solid phase extraction was reported to cause a cartridge block by insoluble lipids [16]. Additionally, electrospray ionization (ESI) may be influenced by the matrix content, as other compounds may compete with the analyte during the ion transfer [30]. Various techniques can be used to mitigate or handle these potential issues.

The analytical method described in this work was developed to be utilized in a pharmacokinetic study, where high concentration of lipids was known to be an inevitable factor that can potentially interfere with the drug measurements. Therefore, we decided to pay special attention to the potential impact of lipid content on the analytical method performance. This attention was reflected in all steps of the method development, refinement and, finally, validation. Thus, the aim of this work was to elaborate an easy, robust, selective and sensitive analytical method for FEN detection by means of HPLC-MS^2^, which would allow for reliable evaluation of this drug concentration in standard and lipemic plasma samples.

## 2. Results

**Figure 1:**
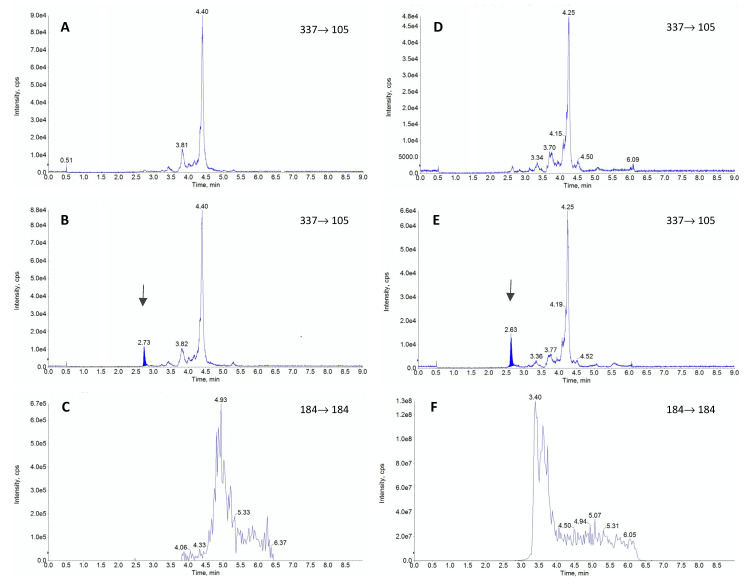
The representative chromatograms obtained during the validation of fentanyl (FEN). The blank sample (**A**), plasma sample containing FEN residues at the limit of quantification (**B**) and glycerophosphocholines (184 *m*/*z*) (**C**) are shown for standard rabbit plasma. For comparison the same chromatograms are presented for lipemic plasma, including blank sample (**D**), sample with FEN addition at the limit of quantification level (**E**) and glycerophosphocholines (**F**). The peak for FEN is seen as a dark-blue shaded area and it’s retention time is indicated by the arrows (ca. 2.6 min).

All the required validation parameters: linearity, repeatability, reproducibility, recovery, selectivity, matrix effect, limit of detection (LOD) and limit of quantification (LOQ) were successfully evaluated and were in the prerequired ranges for both matrices (Table 1). The analysis of 10 blank samples of the matrices did not reveal any interference but visible differences were noted between the standard plasma samples and lipemic samples (Figure 1).

**Figure 2:**
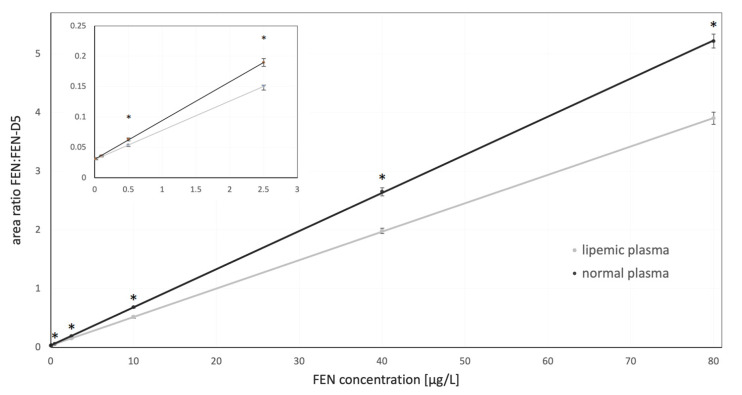
The difference between calibration curves in normal and lipemic plasma samples. The smaller panel represents the lower part of the calibration curves. Vertical bars represent standard deviations associated with the results. The significant differences between the area ratio observed in the two matrices are indicated by asterisks (*p* < 0.01).

In both matrices, the criteria concerning relative retention time were met, and the found analytes corresponded to those of the calibration solutions with a tolerance of ±2.5%. Linearity (R^2^) for the entire examined range (0.02–80 µg/L) was 0.980 and it was identical for both matrices. Initially, the validation included also the concentration of 100 µg/L FEN, but this level had to be removed due to its drastic negative impact on the linearity (R^2^ < 0.95). The two calibration curves were characterized by different equations (Table 1) and visual discrepancies between them can be appreciated in Figure 2. The ratio of the area of FEN, to its internal standard (IS)—fentanyl-D5 (FEN-D5), was different during the two validations. In all high concentrations the differences were significant and only the lowest concentrations, 0.02 and 0.1 µg/L, were similar between the curves.

The recoveries for non-lipemic plasma for all three concentration levels (0.5, 10, 80 µg/L) were high and stable (around 100%), and are reported in Table 2. The same tendency was observed for the IS. Its mean recovery was 110.1 ± 6.1%. In the case of the lipemic matrix, the recovery of FEN depended on the concentration and was approaching the values obtained in the standard matrix only for the highest concentration (Table 3). At the lower levels, the recovery dropped markedly, as indicated in Table 3. The recovery of IS in the lipemic matrix was lower than expected, with a mean value of 43.8%, and presented higher variability (SD 15.8%). The repeatability and reproducibility were always within the acceptance criteria, and no major differences were observed between the two types of the matrix (Table 2 and Table 3). For non-lipemic plasma, the repeatability was lower than 5.9% and within-laboratory reproducibility fell below 6.7%. For lipemic plasma, the repeatability of less than 7.9%, and within-laboratory reproducibility below 8.7% were noted, being only slightly higher than in the normal plasma. The expanded uncertainty was calculated at all seven concentration levels and the values are reported in Table 2 and Table 3. The calculated mean ion suppression of the matrix effects for FEN at the level of 0.5 µg/L was slightly higher for the lipemic plasma (Table 1). Nevertheless, taking into account this result variability, it can be concluded that both matrices exhibit a similar and low level of matrix effect. The screening detection and quantification limits are presented in Table 1.

## 3. Discussion

**Figure 3:**
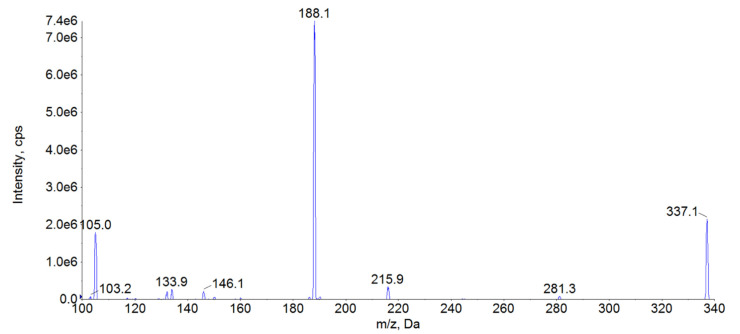
MS^2^ spectra of FEN. The daughter ions with the highest intensity were found to be 105 *m*/*z* and 188 *m*/*z*.

FEN is a synthetic compound belonging to the opioid group, frequently used as an analgesic agent [18]. It exerts an agonistic effect on opioid receptors [18]. The effective plasma concentration for this compound can be as low as 1.0 µg/L, which requires a very sensitive analytical method to adequately measure this drug concentration in the clinical conditions [26]. The most frequent methods used for FEN determination are those based on either liquid chromatography combined with UV-VIS detection [31,32,33,34] or liquid and gas chromatography combined with mass spectrometry [20,24,26,32,35,36,37,38,39,40,41,42,43]. Due to lower sensitivity and precision of UV-VIS detectors, this approach to the drug analysis is notably less frequent [42]. Sensitivity of gas chromatography methods combined with spectrometry is often excellent. However, this approach requires chemical derivatisation and is relatively time-consuming as compared with liquid chromatography [42]. Therefore, determination of FEN is dominated by liquid chromatography-mass spectrometry methods offering a convenient, sensitive and reliable analysis [20,24,26,32,35,36,37,38,39,40,42,44]. For liquid chromatography-mass spectrometry methods different authors consistently reported ESI as the best ionization mode [20,24,26,32,35,36,37,38,39,40,42,44]. Based on the literature data, we also decided to use it in our study. All parameters, namely declustering potential (DP), collision energy (CE), and entrance potential, were optimized with a direct infusion of working standard solution, as shown in Table 4. The ions selected for the analysis (Table 4) are in good accordance with those reported by other authors [20,26,32,35,36,37,38,39,40,42,44,45]. These ions were chosen based on the highest intensity, as indicated by mass spectra for FEN (Figure 3). Contrary to the other reports, we selected 105 *m*/*z* ion as a quantitative ion, despite its lower sensitivity. This choice was motivated by issues related to satisfactory signal linearity at higher concentrations for *m*/*z* 188 ion. The use of the ion with lower sensitivity allowed us to obtain adequate linearity at 80 μg/L. Unfortunately, despite our attempts, extending the method range to 100 μg/L was impossible, as in that case even the selection of the lower intensity ion did not provide satisfactory linearity. As the method was intended to be used during a pharmacokinetic experiment, the upper limit of 80 μg/L was concluded to be sufficient. Also, the obtained LOQ was fully satisfactory for this purpose. Therefore, to address both the necessity for low LOQ and the adequate upper limit of quantification, we selected 0.02–80 μg/L as a concentration range for our method.

The most commonly used columns for FEN separation are C18 columns [20,24,32,36,39,40,42] and biphenyl columns [26,38,44]. One paper documented the use of a pentafluorophenyl column [39], but this selection resulted in a very high matrix effect. The reported length of columns ranged from 50 mm to 150 mm, while the retention time for FEN ranged from 2.2 min [24,32] to 9.1 min [43]. Based on the literature data, we chose a C18 column (Kinetex^®^ 2.6 µm XB-C18 column 50 × 2.1 mm, Phenomenex, Torrance, CA, USA), which provided a relatively short retention time of 2.6 min and very good chromatographic separation of FEN from interferences. The most commonly reported mobile phases comprised 0.1% formic acid in water combined with acetonitrile or methanol in various proportions [20,23,26,32,38,39,40,42,43], but some authors selected ammonium buffer of various concentrations [24,36,38,39,44]. The elution conditions varied between laboratories, and some authors opted for a gradient flow [23,26,36,37,39,44], while others analyzed FEN in an isocratic mode [20,24,32,38,40]. In the current study, we decided to use 0.1% formic acid and methanol in a gradient flow due to the pronounced lipemia in some of the samples, which, according to our and other authors’ opinion [16], may have a significant impact on the final results. Some authors pointed out a considerable matrix effect (>15%), which can significantly affect the results [23,24,37]. For the present method, in addition to a standard investigation of the matrix effect [44], we also monitored phospholipids, which served as a marker of lipid-related impurities in plasma. Based on our experience and the reports of other authors [45,46,47], we monitored the presence of 184→184 ion characteristic for this type of compounds. The presented chromatograms (Figure 1, panel C and F) proved that the used mobile phase and appropriate gradient allowed for determining FEN without any influence of phospholipids. Even lipemic samples were not negatively influenced by the lipid-related impurities as chromatographic conditions allowed to separate them from FEN as seen in the retention time difference (Figure 1, panel F).

The extraction procedure in our study had to be carefully planned, as it was developed to be utilized during pharmacokinetic study where large number of samples had to be efficiently subjected to analysis. The required low level of sensitivity, high throughput of the procedure, cost optimalization and high lipid content in the matrix had to be considered. Based on all these factors and reported literature sources, the liquid-liquid extraction was considered as a best choice to meet all the requirements. A number of different protocols for plasma sample purification for FEN analysis can be found in the literature. Some authors successfully used simple protein precipitation [24,40,42]. Others employed various extraction solutions, like ethyl acetate [48], cyclohexane [34], *n*-butyl chloride [36], *n*-butyl chloride with acetonitrile [20,35], heptane with isoamyl alcohol [49] or heptane with 2-butanol [32]. Our initial tests involved several extraction solutions but the mix of 1-chlorobutyl and acetonitrile provided the best relative recovery. Nevertheless, substantial differences were noted depending on the matrix. In the case of the non-lipemic rabbit plasma, the recovery was very good and slightly exceeded 100%. Taking into consideration that during the extraction the drug was concentrated (300 µL of plasma resulted in 200 µL of the final solution), this result was deemed satisfactory. Other authors reported very similar values after the extraction from plasma or serum [20,32,34,39,41,50]. However, the addition of lipids to the matrix resulted in a significant decrease in the extraction efficacy in the concentration-dependent manner. The highest drug concentration resulted in the most efficient extraction but the lower concentrations did not exhibit a linear decrease in recovery. The probable cause of relatively low recovery for the lipemic matrix was the selection of the extraction solvent and very high lipophilicity of the target compound. In the current study, 1-chlorobutyl and acetonitrile probably could not provide adequate lipid sequestration in the organic layer, as the dielectric constant of 1-chlorobutan is only 7.2 [51]. This resulted in visible, persisting milkiness of the plasma after the extraction. Such inadequate lipid recovery could favor FEN retention in the matrix, since the LogP of this drug is relatively high being 4.05 [50]. As the extraction procedure was initially optimized only for non-lipemic plasma, probably the selection of organic solutions with a lower dielectric constant or prolongation of the extraction procedure could produce better results [52]. Nevertheless, a more efficient transfer of FEN could result in more pronounced interferences with lipids extracted alongside the drug and cause ionization issues and problems with the compound detection. As can be seen in Figure 1, these obstacles were successfully avoided. Thus, the selected extraction provided high sensitivity on the one hand and lack of lipid inference on the other, regardless of the matrix used. Nevertheless, single validation for the drug quantification in both matrices could not be successful as it is clearly depicted in Figure 2. The lipophilic nature of FEN had a significant impact on this issue since the extraction protocol had to balance between effective drug recovery and elimination of lipids in the final extract. For any highly lipophilic drug, finding the compromise between these two aspects would be of utmost importance. However, it should be noted that provided results consider only the selected protocol of liquid-liquid extraction. It is possible that dedicated SPE, solid phase microextraction, QuEChERS or even protein precipitation techniques could provide stable results, unbiased by the excessive presence of lipids. This, however, is out of the scope of this study.

The observed differences in the evaluation of FEN in normal and lipemic plasma showed that uniform evaluation of drug levels in matrices differing in terms of lipid content may lead to substantial errors. Thus, close attention should be paid during routine studies of this drug in the lipemic samples.

## 4. Materials and Methods

### 4.1. Chemicals and Reagents

Analytical grade FEN and FEN-D5 were purchased from Sigma-Aldrich (Darmstadt, Germany). Acetonitrile (J.T.Baker, Gliwice, Poland), 1-chlorobutane (Sigma-Aldrich, Darmstadt, Germany) and ammonia solution 25% (Stanlab, Lublin, Poland) were of HPLC or analytical grade. Formic acid and methanol were obtained from Sigma-Aldrich (Darmstadt, Germany) and were of analytical grade and LC-MS grade, respectively. Ultrapure water was filtered through a Millipore Milli-Q system (Burlington, MA, USA).

### 4.2. Standard Solutions

Stock solutions of 1000 µg/L for FEN and 1000 µg/L for FEN-D5 were prepared volumetrically in methanol and stored at −70 °C for no longer than six months. They were further diluted using methanol:water (50:50 *v*/*v*) solution to prepare working standard solutions for FEN and FEN-D5. The working standard solutions were prepared freshly each day and were used for the construction of standard validation curves.

### 4.3. High Performance Liquid Chromatography Tandem Mass Spectrometry

The analytical system used in the study consisted of an ABSciexExion LC HPLC connected to ABSciex API 5500 Qtrap mass spectrometer (AB Sciex, Concord, ON, Canada). To control the HPLC-MS^2^, the Analyst 1.6.3 software was used and Multiquant 3.2 was employed to process the data. The mass spectrometer was operated in the positive ESI mode with a capillary voltage of 5.5 kV, and the voltage of electron multiplier set to 2.2 kV. The temperature of desolvation was set to 500 °C, gas 1 (air)–40 psi; gas 2 (air)–40 psi; collision gas (N_2_)–medium; nebulizer gas (N_2_)–40 psi; curtain gas (N_2_)–40 psi. The stationary phase was a Kinetex^®^ 2.6 µm XB-C18 column 50 × 2.1 mm (Phenomenex, Torrance, CA, USA), equipped with a SecurityGuard™ ULTRA C18 2.1 mm precolumn (Phenomenex, Torrance, CA, USA), and it was working at a constant temperature of 40 °C. The mobile phase was composed of two solutions: A (0.1% formic acid) and B (methanol) in a gradient mode that started with 5% of B. From 0.5 to 1 min, the concentration of B was raised to 30%, then from 1 to 2 min the concentration of B was raised to 50%, then from 2 to 3 min the concentration of B was raised to 90%, and left for 2.2 min. Finally, B concentration was decreased from 5.2 to 6 min to 5% and left for 3 min. The flow rate was 0.4 mL/min. The ions were monitored in MS^2^ mode, and the precursor and the daughter ions of FEN, as well as parameters selected for IS, are shown in Table 4. Finally, 105.0 *m*/*z* ion was used as a quantitative ion and 188.0 *m*/*z* was used as a qualitative ion.

### 4.4. Validation Procedure

Due to the reduced signal of the IS in the lipid-rich matrix noted during the exploratory analysis, we decided to conduct a separate validation for this type of the matrix. Thus, all mentioned validation criteria were separately applied to the normal plasma samples and the lipid-enriched plasma. Validation of the analytical method was carried out according to the International Council of Harmonization Q2 (R1) method validation guidelines [53]. This selection was based on a common usage of this procedure for validation in the medical and toxicological analysis [47,48,54]. Selectivity, linearity, uncertainty, precision, LOD and LOQ were evaluated during this process. Analyte standard solutions at 0.02; 0.1; 0.5; 2.5; 10; 40; 80 µg/L containing IS FEN-D5 (10 µg/L), were subjected to the liquid-liquid extraction as described in the following paragraph and the HPLC-MS^2^ analysis was conducted. The analyte peak area was plotted against the corresponding concentrations and the calibration curves were set up employing the least-squares method. Similarly to other works, LOD and LOQ were estimated by calculations based on the signal-to-noise ratio [20,28,48]. This determination was performed by comparing the measured signals from the samples with known low concentrations of the analyte with those of blank samples and establishing the minimum concentration at which the analyte can be reliably detected or quantified. A typical signal-to-noise ratio was selected as 3:1 for LOD and 10:1 for LOQ [20,28,48]. The LOQ level was included in the validation curves, and all validation parameters have been calculated also for this concentration. Repeatability and reproducibility were determined at seven concentration levels (six samples of each level for each matrix) 0.02; 0.1; 0.5; 2.5; 10; 40; 80 µg/L. For repeatability, the samples were analyzed by the same operators, on the same day with the same instrument and were calculated as the relative standard deviation (RSD, %). For within-laboratory reproducibility, the other two sets of blank samples were fortified and analyzed by different operators, on two different days, with the same instrument, and were calculated as the relative standard deviation (RSD, %). The expanded uncertainty was calculated at the same concentration levels, by applying a coverage factor of 2, which gave the level of confidence of approximately 95% [55]. In the selectivity study, possible interferences with matrix components were checked based on the analysis of 10 blank samples, originated from different animals, for each kind of the matrix, separately for standard plasma and lipemic samples. The matrix effect was checked by analyzing five different samples at 2.5 µg/L and calculated using the equation proposed by Matuszewski [44]. The drug recovery was calculated by comparing the signal produced by the drug after standard extraction procedure with that obtained after the addition of the drug to the extracted blank matrix [56]. This protocol was used to assess the recovery of FEN at 0.5, 10, and 80 µg/L, and IS at 10 µg/L. The assessment was conducted in triplicate. Since the normal distribution was not confirmed in all sets of measurements, the data was statistically analyzed using the Mann–Whitney test, and *p* < 0.05 was considered as indication of significant difference. R (version 4.0.3, The R Foundation, Vienna, Austria) and RStudio (version 4.1.0, RStudio, Boston, MA, USA) softwares ware used to perform necessary calculations.

### 4.5. Matrix Collection and Preparation

Plasma samples used in the method development and validation were obtained from rabbits involved in the pharmacokinetic/pharmacodynamic study and were the by-products associated with necessary procedures. The experiment was approved by the Local Animal Experimentation Committee in Wrocław, permit number 42/2017. The plasma originated from animals that did not receive any drugs except for isoflurane used for inhalation anesthesia. The plasma obtained under such conditions was treated as a normal (non-lipemic) plasma.

**Figure 4:**
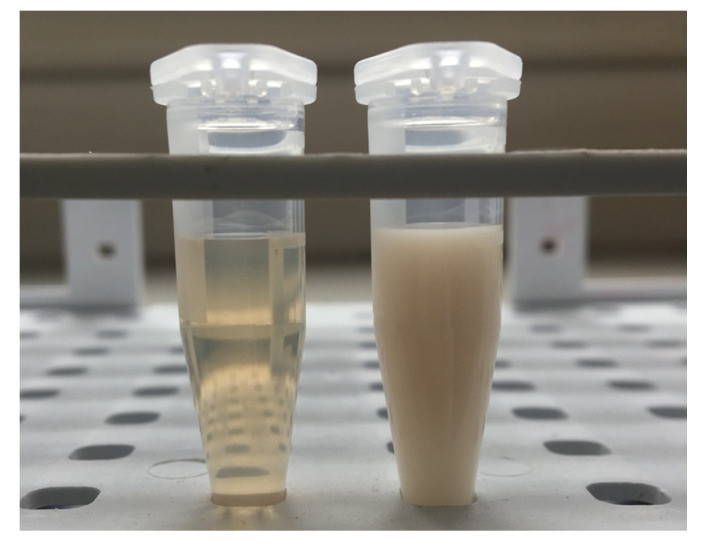
Apparent visual difference between the two matrixes used in the study, the normal rabbit plasma (on the **left**) and artificially produced lipemic samples obtained by Intralipid^®^ addition to the normal plasma (on the **right**).

However, it was impossible to collect the amount of lipemic plasma required for the validation directly from the animals. Thus, to acquire a reasonable replacement, the normal rabbit plasma was supplemented with Intralipid 20%^®^ (Fresenius Kabi, Uppsala, Sweden) as a substitution for the lipemic samples. Intralipid^®^ is a lipid emulsion used for parenteral nutrition under clinical conditions and its composition is very close to the chylomicrons observed in blood during postprandial lipemia [54]. Thus, it was decided that this product can reliably mimic the real-life lipemic samples. Additionally, to secure stable and high levels of lipemia, it was decided that lipid addition to the concentration of 20 mmol/L of triglycerides should be adequate. This assumption was based on our preliminary findings in rabbits after administration of Intralipid^®^ as a lipid rescue therapy (unpublished data). This level resulted in clearly visible milky plasma appearance as can be appreciated in Figure 4.

### 4.6. Sample Extraction Procedure

Three hundred microliters of blank rabbit plasma or plasma fortified with FEN standard were added to 50 µL of FEN-D5 solution. The concentration of IS was 60 µg/L, and so the final concentration in the plasma was 10 µg/L. Then, 50 µL of ammonia solution were added, and the samples were manually shaken. The extraction solution was composed of 1-chlorobutyl:acetonitrile in proportion 4:1 (*v*/*v*) and 1000 µL of the solution were added to the sample. The extraction was conducted for 15 min on a horizontal shaker. After centrifugation, 800 µL of the extract were transferred into fresh tubes and evaporated until dry in a vacuum concentrator (Eppendorf, Hamburg, Germany; vacuum mode, 30 °C). The residues were redissolved in 200 µL of methanol and filtered through Nanosep MF with a 0.2 µm nylon membrane (Pall Corporation, Puerto Rico, PR, USA). An identical extraction procedure was used for both tested types of matrices.

## Figures and Tables

**Table 1:** Validation report for fentanyl.

Matrix	LOD [µg/L]	LOQ [µg/L]	Matrix Effect (%)	Concentration Range (µg/L)	Determination Coefficient	Calibration Curve
normal plasma	0.005	0.015	4.0 ± 2.6%	0.02–80	0.980	y = 0.065x + 0.03
lipemic plasma	0.008	0.02	6.1 ± 3.6%	0.02–80	0.980	y = 0.0485x + 0.03

**Table 2:** Parameters obtained for the calibration curves during validation of normal (non-lipemic) plasma.

Level	Repeatability (RSD_r_,%) (*n* = 6)	Within-Lab Reproducibility (RSD_wR_,%) (*n* = 18)	Expanded Uncertainty (µg/L)	Recovery [%]
0.02 µg/L	5.9 ± 6.1	6.7 ± 4.8	0.02 ± 0.006	-
0.1 µg/L	4.8 ± 4.6	5.6 ± 4.4	0.1 ± 0.03	-
0.5 µg/L	4.6 ± 3.0	4.8 ± 4.1	0.5 ± 0.12	107.7 ± 1.4
2.5 µg/L	4.4 ± 3.1	4.2 ± 4.6	2.5 ± 0.60	-
10 µg/L	2.8 ± 2.6	3.4 ± 3.2	10 ± 2.10	111.1 ± 2.5
40 µg/L	4.2 ± 3.4	4.7 ± 3.6	40 ± 12.8	-
80 µg/L	3.7 ± 2.9	4.9 ± 3.1	80 ± 18.3	103.6 ± 9.5

RSD-relative standard deviation.

**Table 3:** Parameters obtained for the calibration curves during validation of lipemic plasma.

Level	Repeatability (RSD_r_,%) (*n* = 6)	Within-Lab Reproducibility (RSD_wR_,%) (*n* = 18)	Expanded Uncertainty (µg/L)	Recovery [%]
0.02 µg/L	7.9 ± 5.1	8.7 ± 4.8	0.02 ± 0.008	-
0.1 µg/L	5.8 ± 4.3	6.6 ± 4.2	0.1 ± 0.03	-
0.5 µg/L	4.4 ± 3.6	4.4 ± 4.3	0.5 ± 0.14	46.4 ± 5.0
2.5 µg/L	4.1 ± 3.0	4.2 ± 4.1	2.5 ± 0.50	-
10 µg/L	3.4 ± 2.6	3.9 ± 3.8	10 ± 2.20	32.0 ± 4.8
40 µg/L	3.2 ± 4.6	5.3 ± 3.3	40 ± 13.4	-
80 µg/L	3.5 ± 23.9	4.3 ± 3.5	80 ± 20.7	73.5 ± 7.7

RSD-relative standard deviation.

**Table 4:** The description of parameters used for FEN determination.

Analyte	Precursor Ion (*m*/*z*)	Ion Transition (*m*/*z*)	Declustering Potential (eV)	Entrance Potential (eV)	Colision Energy (eV)
Fentanyl	337.0	188.0 105.0	101 101	10 10	29 43
Fentanyl-D5	342.0	188.0	101	10	29

## Data Availability

The data is available upon request from the corresponding author.

## Sample Availability

Samples of the compounds are not available from the authors.
